# Epigenetic silencing of EphA1 expression in colorectal cancer is correlated with poor survival

**DOI:** 10.1038/sj.bjc.6604970

**Published:** 2009-03-10

**Authors:** N I Herath, J Doecke, M D Spanevello, B A Leggett, A W Boyd

**Affiliations:** 1 Leukaemia Foundation Research Laboratory, The Queensland Institute of Medical Research, 300 Herston Road, Brisbane, Queensland 4029, Australia; 2 Cancer and Population Studies, The Queensland Institute of Medical Research, 300 Herston Road, Brisbane, Queensland 4029, Australia; 3 Conjoint Gastroenterology Laboratory, Clinical Research Centre, Royal Brisbane Hospital Research Foundation, 300 Herston Road, Brisbane, Queensland 4029, Australia; 4 Department of Medicine, The University of Queensland, Brisbane, Queensland 4029, Australia

**Keywords:** EphA1, colorectal cancer, methylation, quantitative PCR, downregulation

## Abstract

Aberrant expression of Eph and ephrin proteins has well-established functions in oncogenesis and tumour progression. We describe EphA1 expression in 6 colorectal cancer (CRC) cell lines, 18 controls and 125 CRC specimens. In addition, a well-characterised cohort of 53 paired normal colon and CRCs was also assessed. Expression of EphA1 mRNA was assessed by quantitative real-time PCR and correlated with protein expression by flow cytometry, immunoprecipitation, western blotting and immunohistochemistry. Significant upregulation (2- to 10-fold) of EphA1 was seen in over 50% of cases (*P*=0.005) whereas many of the remainder showed downregulation of EphA1. Intriguingly, EphA1 over-expression was more prevalent in stage II compared to stage III CRCs (*P*=0.02). Low EphA1 expression significantly correlated with poor survival (*P*=0.02). Epigenetic silencing appeared to explain the loss of EphA1 expression as methylation of the EphA1 CpG island strongly correlated with low EphA1 expression (*P*<0.01). Furthermore, EphA1 re-expression could be induced by treatment with demethylating agents. Our findings identify EphA1 as a potential prognostic marker in CRC. Although therapies targeting high EphA1 expression seem plausible in CRC, the loss of expression in advanced disease suggests a potential risk that targeted therapy, by selecting for loss of expression, might contribute to disease progression.

The 16 vertebrate Eph receptors (EphA1–10, EphB1–6) form the largest subfamily of receptor tyrosine kinases. Activation of signalling by these receptors is mediated by interaction with nine cell-surface counter receptors known as ephrins. Eph receptors are divided into two classes, A and B, based on structural features of their ligand-binding domains and preferential binding to either ephrins A1–6 or ephrins B1–3 groups, respectively (Boyd and Lackmann, 2001). The ephrin A ligands are bound to the cell surface by a glycosyl phosphatidylinositol anchor and bind to class A receptors, whereas the ephrin B ligands are type I transmembrane proteins and bind to class B receptors (Gale *et al*, 1996).

Eph–ephrin interactions are known to mediate both pro-adhesive and anti-adhesive signals in both normal and tumour cells and also have a function in tumour angiogenesis (Adams, 2002; Wilkinson, 2003). High Eph and ephrin expression has been reported in many human cancers including various carcinomas, melanoma, sarcoma, kidney and brain tumours (Dodelet and Pasquale, 2000; Nakamoto and Bergemann, 2002; Brantley-Sieders *et al*, 2004). EphB and ephrin B proteins have been implicated in both normal and malignant epithelial tissues. Signalling through EphB2/B3 and ephrin B ligands regulates cell sorting in the mature gut epithelium (Batlle *et al*, 2002; Sancho *et al*, 2003). Over-expression of EphB2, EphB4 and ephrin B1 has been described in gastric, colon and breast cancers (Stephenson *et al*, 2001; Berclaz *et al*, 2002; Kataoka *et al*, 2002). Interestingly, over-expression of ephrin B2 in colorectal cancers (CRCs) appears to correlate with increased tumour angiogenesis, unexpectedly resulting in reduced tumour growth as new vessels were malformed (Liu *et al*, 2002, 2004).

Although the EphA/ephrin A system has also been implicated in epithelial tissue structure and function, there is less data on EphA/ephrin A expression in gut and CRC (Maru *et al*, 1990; Saito *et al*, 2004). EphA1, originally isolated as an amplified gene in a carcinoma cell line (Hirai *et al*, 1987), is preferentially expressed in epithelial cells. Ephrin A1 is the highest affinity binding ligand for EphA1 (Coulthard *et al*, 2001), although it also binds ephrin A3 and A4 with lower affinity. In mice, EphA1 is expressed in many epithelial tissues including skin, kidney, liver and thymus (Coulthard *et al*, 2001). Over-expression of human EphA1 has been observed in prostate, gastric and colon carcinomas (Hirai *et al*, 1987; Maru *et al*, 1990; Robinson *et al*, 1996; Kao *et al*, 2003). More recently, significant downregulation of EphA1 was reported in non-melanoma skin cancers (Hafner *et al*, 2006).

Despite a few reports of aberrant expression of EphA1 in CRCs, the prevalence of EphA1 expression and its potential prognostic and therapeutic function in CRC has not been examined systematically. We describe the expression of EphA1 in a series of CRC cell lines, clinical CRC samples and a well-characterised cohort of paired normal and CRC samples using quantitative real-time PCR. We show significant EphA1 over-expression in CRCs compared to controls. Interestingly, investigation of paired normal and CRC samples revealed significant EphA1 over-expression in locally invasive CRC (stage II) but also downregulation in metastatic CRC (stage III). Further, there is evidence for active selection against expression in metastatic lesions, mediated through epigenetic gene silencing. Intriguingly, low EphA1 expression correlated with shortened survival. The potential consequences of this on colon cancer cell biology are discussed.

## Materials and methods

### Cell lines and clinical samples

Six CRC cell lines (LIM1215, CaCo2, LISP-1, LOVO, HCT116 and HT29) were cultured in RPMI 1640 medium with 10% fetal bovine serum (FBS). CaCo2 was cultured in Dulbecco's modified medium (Gibco, Mount Waverley, VIC, Australia) with 20% FBS. Lines were maintained in a 5% CO_2_ humidified incubator at 37°C. A total of 196 colon specimens were assessed in this study, including colon tissue qPCR arrays (OriGene Technologies, Rockville, MD, USA) consisting of 143 cDNA samples and a well-characterised cohort of 53 paired normal and CRC specimens. The qPCR arrays contained cDNA from 18 normal colon, 21 stage I, 40 stage II, 43 stage III and 21 stage IV CRC samples. The median age of the patients was 70 years (range 31–93 years) with a male/female ratio of 73 : 70. The 53 paired tumour and adjacent non-malignant samples were obtained from the Princess Alexandra Hospital tissue bank and the Royal Brisbane Hospital (Brisbane, Australia). Stages II and III were predominant in this cohort with only four stage I and three stage IV cancers (Supplementary Table 1). The median age of the patients was 72 years (range 29–84 years) with a male/female ratio of 28 : 25. Additional clinical details are presented in Supplementary Table 1. All patient samples were obtained after specific informed consent procedures were approved by the institutional ethics committees of the relevant institutions.

### RNA extraction and cDNA synthesis

Total RNA was isolated using the RNeasy Mini kits (Qiagen, Doncaster, VIC, Australia), according to the manufacturer's instructions. RNA quality was assessed by agarose gel electrophoresis. Before cDNA synthesis, samples were treated with RQ1 RNase-free DNase I (Promega, Sydney, NSW, Australia) and first strand cDNA was synthesised by reverse transcription using Superscript III Reverse Transcriptase (Invitrogen, Mount Waverley, VIC, Australia), according to the manufacturer's instructions.

### Relative quantitation by real-time PCR

Protocol for real-time PCR is described in Supplementary methods.

### Immunoprecipitation and western analysis

Protocols for immunoprecipitation and western analysis are described in Supplementary methods.

### Immunohistochemistry

The protocol for immunohistochemistry is described in Supplementary methods.

### Methylation analysis

#### Sodium bisulfite modification

Genomic DNA was subjected to sodium bisulfite modification using the CpGenome DNA modification kit (Chemicon, Sydney, NSW, Australia) to convert the unmethylated cytosines to uracil.

#### Bisulfite genomic sequencing

Methylation status of the 5′-CG-rich region and the EphA1 CpG island was determined through bisulfite sequencing and melt curve analyses. EphA1 5′ UTR primers were: 5′-TTTAAGGAGGTGAATTAGGTGA-3′ (sense) and 5′-CCATAACTCCGAACCGAAAC-3′ (antisense). PCR was conducted in a final volume of 25 *μ*l including 1 × PCR buffer, 80 ng of bisulfite-modified DNA, 3 mM Mg^2+^, of each primer, 3.2 mM dNTP and 2 M betaine. PCR products were generated using a touchdown PCR cycle with annealing temperatures decreasing 0.5°C per cycle from 68 to 60°C.

For the EphA1 CpG island, bisulfite-modified genomic DNA samples were amplified using double nested oligonucleotides (the primer sequences were designed based on the http://www.mdanderson.org/leukemia/methylation/bpcr.html website). These primer sets were specific to modified templates with no CpG sites in their sequences, therefore, both methylated and unmethylated templates were amplified. EphA1 CpG island primers were F1 – 5′-GGTGTTGGTTTTTGGGGTTA-3′ and R1 – 5′-AAAATTCCCTCCCCACTCC-3′. Nested primers were F2 – 5′-GGTTAGGGTTGGTGTTGTTGTT-3′ and R2 – 5′-AAAACCAAAAATAAACCTAACAAT-3′. PCR was performed in a final volume of 25 *μ*l including 80 ng of bisulfite-modified DNA, 10 × buffer, 3 mM Mg^2+^, 0.4 *μ*M of each primer, 3.2 mM dNTP and 2 M betaine. Initial amplification was carried out using touchdown PCR from 68 to 60°C. The second round was performed in a final volume of 25 *μ*l with 2.5 *μ*l of the primary PCR template as described above. PCR was conducted at 95°C for 30 s, 50°C for 30 s and 72°C for 30 s for 35 cycles followed by a final extension at 72°C for 10 min.

Twenty paired normal and CRC samples for the 5′-CG-rich region and 37 paired normal and CRC samples for the CpG island were screened through sequencing. PCR products were cloned into the pGEM-T Easy vector (Promega) following agarose gel purification and 10 individual clones were sequenced from each sample. The percentage of methylation was calculated by dividing the number of CpG sites methylated with the total number of CpG sites assessed.

#### In-tube DNA melting profile

A total of 21 paired normal and CRC samples were assessed by fluorescence melting curve analysis as described previously (Worm *et al*, 2001). DNA with no methylation of the target gene and DNA with complete methylation (using *Sss1* methyltransferase to methylate all cytosine residues) were used as negative and positive controls, respectively. Of the 21 paired normal and CRC samples, 5 were also subjected to bisulfite sequencing to ensure that the results were comparable.

PCR products were generated using a block thermocycler as described previously. PCR product (15 *μ*l) was mixed with 5 *μ*l of H_2_O and 10 *μ*l of Quantitect SYBR Green PCR Master Mix (Qiagen). DNA melting curves were acquired on the Rotor-Gene 3000 by measuring the fluorescence of SYBR Green I during a linear temperature transition from 70 to 95°C with a ramp of 0.5°C s^−1^.

### *In vitro* demethylation and histone deacetylase inhibition

To assess whether EphA1 expression could be restored, we treated cell lines with a methyltransferase inhibitor with or without a histone deacetylase (HDAC) inhibitor. A total of 4 × 10^5^ cells were plated into Petri dishes and treated with freshly prepared 2 *μ*M 5′-aza-2′-deoxycytidine (Sigma, Castle Hill, NSW, Australia) with or without the HDAC inhibitor, suberic bishydroxamate (SBHA) at a final concentration of 30 *μ*g ml^−1^. Cells were treated for periods of 24, 48 and 72 h with media and inhibitors replaced every 24 h. Cells were harvested at approximately 80% confluence after the completion of the treatments. Quantitative PCR and bisulfite sequencing were performed using RNA and DNA, respectively, as described above. Flow cytometry was used to confirm protein expression. All drug assays were performed in duplicate for reproducibility.

### Flow cytometry

The protocol for flow cytometry is described in Supplementary methods.

### Statistical analysis

Quantitative gene expression data from tissue arrays and paired normal and CRC samples were initially analysed using the non-parametric Mann–Whitney *U*-test and Kruskal–Wallis test. To enable the adjustment for covariates such as age and gender, we transformed expression data into quartiles and analysed using binary logistic regression (response variable: tumour/no tumour). Expression data from paired normal and CRC samples were also analysed using the Kaplan–Meier and Cox proportional hazards methods. Final statistical models were adjusted for patient characteristics such as age, gender, type, site and stage. All statistical analyses were performed using SPSS for Windows version 15.0, and a *P*-value of <0.05 was considered statistically significant. Where appropriate, a Bonferroni adjustment was applied to *P*-values.

## Results

### EphA1 expression in CRC cell lines

**Figure 1:**
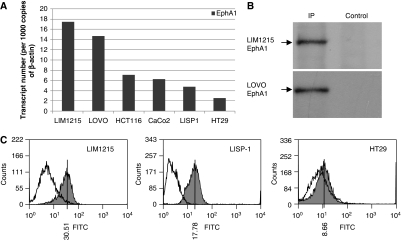
EphA1 mRNA and protein expression in colorectal cancer (CRC) cell lines. (**A**) EphA1 transcript levels normalised to 1000 copies of *β*-actin quantitated by qPCR. (**B**) EphA1 protein expression in LIM1215 and LOVO cell lines determined through immunoprecipitation followed by western blot analysis using an in-house rabbit anti-EphA1 antibody. (**C**) EphA1 protein levels determined by flow cytometry analysis. Fluorescence resulting from EphA1 protein expression (shaded) is shown relative to the secondary-only control cells. The median fluorescence value is shown.

Quantitative PCR analysis revealed a diversity of EphA1 mRNA expression in CRC cell lines, with lines showing high (LIM1215, LOVO), moderate (CaCo2, HCT116, LISP1) and low expression (HT29) (Figure 1A). Protein expression was examined by immunoprecipitation and western blotting. EphA1 was expressed as a single protein of the expected size (∼120 kDa) (Figure 1B) and was shown to correlate well with mRNA expression. The correlation of mRNA with protein expression was further confirmed by analysis of EphA1 protein by flow cytometry (Figure 1C).

### EphA1 expression in colon tissue qPCR arrays

**Figure 2:**
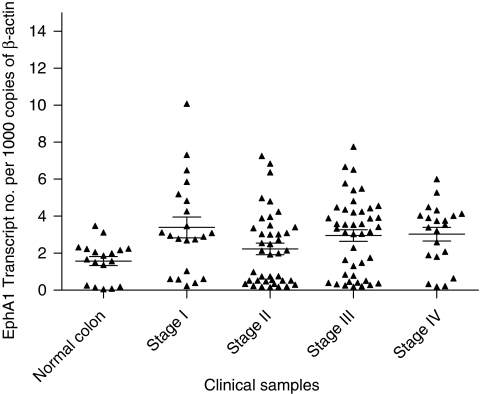
EphA1 mRNA expression in quantitative PCR (qPCR) colon tissue arrays. EphA1 transcript levels normalised to 1000 copies of *β*-actin. The horizontal bars represent the mean and the standard error of the mean.

In keeping with the cell line data, heterogeneous EphA1 expression was also observed in CRC samples. Overall, despite this heterogeneity, when the pooled CRC results were compared with normal colon specimens, EphA1 expression was significantly higher in CRCs compared to the controls (*P*=0.005) (Figure 2). Interestingly, this difference was only apparent in men (*P*=0.003). Using binary logistic regression to estimate the risk of normal control tissues progressing to a CRC, we identified an approximate twofold rise in risk per quartile increase in EphA1 expression (*P*=0.01, OR=1.94, CI=1.19–3.40).

Although a majority of CRCs showed upregulation of EphA1, a cohort of CRCs also demonstrated downregulation compared to normal colon specimens (Figure 2). No correlations were seen between EphA1 expression, stage, grade or age.

### EphA1 expression in CRC clinical samples

**Figure 3:**
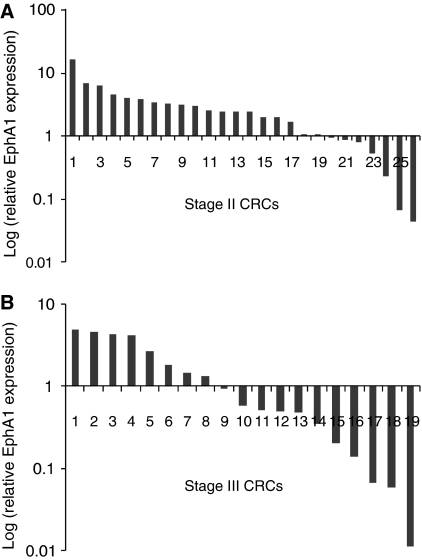
Gene expression level of EphA1 in (**A**) stage II and (**B**) stage III colorectal cancer (CRC) samples. Expression is shown relative to its paired normal sample.

**Figure 4:**
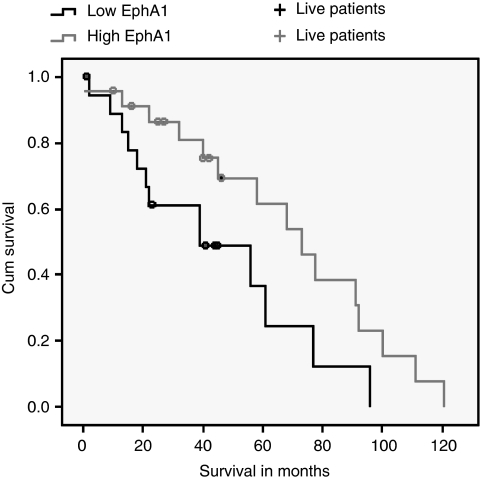
Unadjusted Kaplan–Meier survival curves from the date of diagnosis for patients with high (↑) and low (↓) levels of EphA1 expression. Follow-up time, from the date of diagnosis to the date of last review for patients still living, is represented by the + symbol.

To further examine the heterogeneity of EphA1 expression, we analysed 53 cases in which both normal and CRC tissues had been obtained from each patient. Again, heterogeneous EphA1 expression was observed, with 2- to 10-fold upregulation compared with paired normal colon in 52% of tumours. Interestingly, markedly reduced levels of EphA1 expression compared with paired normal colon were observed in 42% of CRCs. Of these CRCs, 10 cases were more than 5- to 10-fold downregulated compared to the paired normal control. Intriguingly, EphA1 downregulation was significantly more common in stage III compared to stage II CRCs, most of which over-expressed EphA1 mRNA (*P*=0.02) (Figure 3). Patients with low EphA1 expressing CRCs had significantly lower survival than those with high EphA1 expressing CRCs after adjusting for age, gender and stage (*P*=0.02, RR=1.7, 95% CI=1.0–2.8) (Figure 4). EphA1 expression was independent of age, gender, tumour type and grade, although overall survival was marginally better in women compared to men (*P*<0.05).

### Immunohistochemistry

Immunohistochemistry for EphA1 was assessed in the 53 CRCs and paired normal tissue. In normal colonic epithelium, stronger membrane staining was observed at the surface epithelium and was absent at the base of the crypt. In the corresponding CRCs, membrane EphA1 expression was observed with varying degrees of cytoplasmic expression (Supplementary Figure 1). Overall, immunohistochemistry data showed a good correlation with EphA1 mRNA expression in the clinical samples (Supplementary Table 3).

### EphA1 promoter methylation inversely correlates with EphA1 expression in CRC

Although many reports (Hirai *et al*, 1987; Maru *et al*, 1990; Robinson *et al*, 1996; Kao *et al*, 2003) have shown upregulation of EphA1 in CRCs, and more recently downregulation, mechanisms of downregulation have not been examined (Hafner *et al*, 2006). In seeking a mechanism for EphA1 downregulation, epigenetic silencing due to methylation analysis of a CpG island in the *EphA1* gene was investigated.

**Figure 5:**
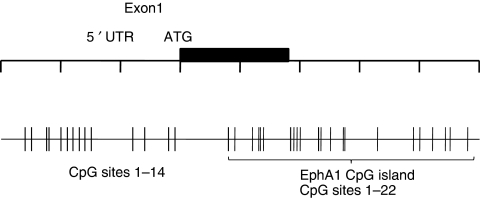
Genomic structure and localisation of the CpG regions of EphA1. The CpG sites are indicated by vertical bars. The methylation status of the 22 CpG sites within the CpG island and the 14 CpG sites located in the CpG-rich 5′-CG-rich region were assessed using bisulfite sequencing.

Analysis of the human *EphA1* gene on the http://www.ebi.ac.uk/emboss/cpgplot/ website using the accepted definition of a CpG island (⩾200 bp with a C+G content >50% and an observed CpG/expected CpG >0.6) (Gardiner-Garden and Frommer, 1987) was performed (Supplementary Figures 2 and 3). We identified a 251 bp CpG island starting 13 bp downstream of the translation start site (A of the ATG=+1) that spans exon 1 and intron 1 of the *EphA1* gene and contained 22 CpG sites (Figure 5). A 153 bp CG-rich region immediately upstream of the translation start site, encompassing the 5′ UTR region and proximal promoter region, contained 14 CpG sites but did not satisfy the criteria of a CpG island.

Initial screening of the 5′-CG-rich region through bisulfite sequencing revealed consistent methylation of CpG sites 12–14 (Figure 5) in all CRC and corresponding normal samples. Methylation of these sites was present regardless of the level of EphA1 mRNA expression, suggesting that this region is not involved in gene regulation. However, this provided a useful internal control for assessment of the effect of methylation on gene expression in this region.

Preliminary sequencing of the CpG island using bisulphite-treated DNA from 37 paired normal and CRC samples established that the average rate of methylation in these samples was 20.4%. We employed melting profile analysis as a means to assay for methylation status. To establish a baseline, we used the CRC cell line LIM1215 expressing high levels of EphA1. Bisulfite sequencing of LIM1215 established that there was no detectable methylation of the EphA1 CpG island in this cell line. Conversely, when treated with *Sss*1 methyltransferase, the EphA1 CpG island was completely methylated in this cell line. These samples were used as negative and positive controls in each run (Supplementary Figure 4A). Using a dilution series, it was established that the presence of greater than 20% methylation results in a shift of the curve (Supplementary Figure 4B). On the basis of the average rate of methylation observed through bisulfite sequencing data and melt curve analysis, we found that a case was considered methylated if the level of methylation was greater than 20% and unmethylated if the level was less than 20%.

Methylation of EphA1 was observed in 26 out of 53 (49%) of CRCs. Methylation was also detected in 15 cases of non-malignant colon tissue. No methylation was detected in 21 out of 53 (40%) cases. In keeping with a function in silencing gene expression, methylation was more widespread in patients displaying downregulation of the gene (*P*<0.01) (Supplementary Figure 5A). Intriguingly, methylation was significantly higher at the 3′ end of the CpG island (CpG sites 11–22) compared to the entire CpG island (*P*<0.01) (Supplementary Figure 5B). An inverse correlation was identified between overall methylation and EphA1 expression (*r*=−0.3); however, the correlation coefficient was stronger when compared to the 3′ end of the CpG island (*r*=−0.6). A strong positive correlation was also evident between increased EphA1 expression and the absence of methylation (*r*=0.7).

Our studies did not attempt to elucidate whether loss of EphA1 expression was due to biallelic methylation or a combination of methylation and allelic loss. Further examination of bisulfite sequences of the few cases over-expressing EphA1 in the presence of methylation revealed that methylation was primarily present at the 5′ end of the CpG island and not the 3′ end of the island where methylation was primarily detected in low expressing CRCs.

### 5′-Aza-2′-deoxycytidine treatment and SBHA treatment of CRC cell lines

**Figure 6:**
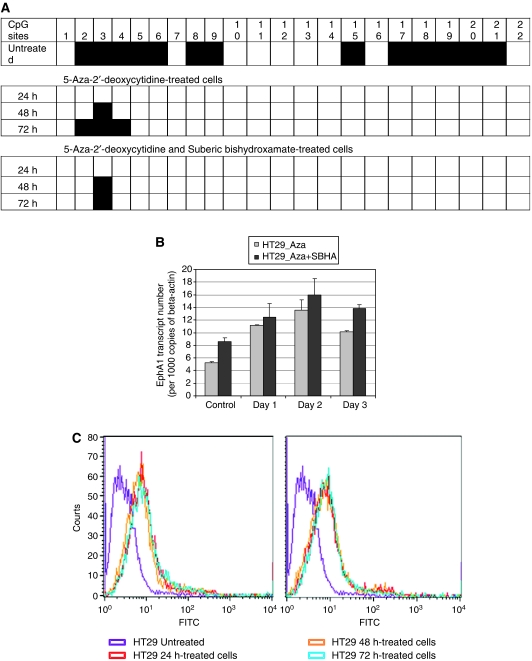
Correlation between methylation status and EphA1 expression. HT29 cells were treated with 5′-aza-2′-deoxycytidine and histone deacetylase inhibitor (suberic bishydroxamate, SBHA) for 24, 48 and 72 h. (**A**) Methylation status of the EphA1 CpG island following treatment as verified by sequencing. (**B**) EphA1 mRNA expression quantitated by real-time PCR following treatment. (**C**) EphA1 protein levels determined by FACS analysis following treatment.

Direct evidence that methylation influences EphA1 expression was obtained by demonstrating that 5′-aza-2′-deoxycytidine treatment restored expression in HT29, and increased expression in LISP1 and HCT116 cells (Figure 6 – data shown for HT29). EphA1 mRNA expression, methylation status and protein expression were examined through qPCR, bisulfite sequencing and flow cytometry, respectively. Following 5′-aza-2′-deoxycytidine treatment, EphA1 underwent complete demethylation at CpG sites 1–22 (Figure 6A) and was accompanied by re-expression of the gene (Figure 6B and C). Because methylated DNA binds methylcytosine binding proteins that in turn interact with HDAC, the cells also were treated with the HDAC inhibitor, SBHA, in combination with the demethylating agent. Bisulfite sequencing revealed some aberrant demethylation in samples treated with SBHA alone (data not shown); however, this did not result in complete demethylation of the sample as with 5′-aza-2′-deoxycytidine. Combination treatment resulted in a further increase in gene expression; however, this was not significantly higher compared to the demethylating agent alone.

## Discussion

In this study we show that human CRC cell lines have variable levels of EphA1 expression. To validate the use of qPCR in tumour samples, we used these lines to show that mRNA expression correlated closely with protein expression (Figure 1A and B). When 125 CRC samples were compared with a set of 18 normal colon samples a significant increase in EphA1 expression was seen in the majority of CRC samples. However, all stages contained some samples with reduced expression compared with the normals. To make a more precise comparison of tumour with normal tissue, we obtained 53 paired normal and malignant CRCs cases. The heterogeneity of expression was confirmed in these samples with over half showing significantly increased expression compared with paired normal and most of the remainder showing significantly decreased expression compared to paired normal. As with cell lines, EphA1 mRNA levels correlated with protein levels as determined by immunohistochemistry. In seeking to understand this biphasic expression pattern, we found that reduced EphA1 expression was more frequent in late-stage CRCs (stage III *vs* stage II), suggesting that there was a selective loss of expression during tumour progression. In support of this notion, patients with low EphA1 expressing tumours had significantly shorter survival than the high EphA1 group.

We had previously shown that reduced expression of EphA3 in haematological tumours was linked to promoter methylation (Dottori *et al*, 1999). As with EphA3, the *EphA1* gene was shown to have a CpG island encompassing the 5′ end of the gene. We showed that the CpG island was significantly hypermethylated in low EphA1 expressing CRCs. Methylation of the 3′ end of the CpG island was even more significant. The re-expression of EphA1 upon treatment with a demethylation agent suggests that methylation has a regulatory function in EphA1 expression. Conservation of EphA1 across species raise the possibility that this 3′ region of the CpG island harbours key regulatory elements of the *EphA1* gene. Notwithstanding contributions by other mechanisms, such as loss of heterozygosity, these data indicate that control of gene expression by methylation is a major mechanism of silencing of EphA1.

Our data show that EphA1 over-expression is commonly seen in locally invasive CRC but that downregulation is more frequent in metastatic CRC, in many cases mediated through epigenetic gene silencing. A similar phenomenon has been described for EphB2, where the loss of expression was associated with cancer progression (Batlle *et al*, 2005; Lugli *et al*, 2005; Guo *et al*, 2006), and higher EphB2 expression was associated with prolonged survival (Guo *et al*, 2006). More recently, EphB2 was reported to be inactivated through promoter hypermethylation in a subset of CRCs (Alazzouzi *et al*, 2005). EphB1 downregulation was also observed in pooled CRCs (Hafner *et al*, 2004). Similarly, there is emerging evidence to suggest that EphA RTK may also be downregulated in other malignancies. A recent study demonstrated significant downregulation of EphA1 in basal cell and squamous cell carcinomas, through immunohistochemistry (Hafner *et al*, 2006). Kataoka *et al* (2004) demonstrated that EphA2 and ephrin A1 expression in CRC was associated with clinicopathological parameters. Both genes were significantly over-expressed in stages I and II; however, this was less apparent in stages III and IV, suggesting that loss of these genes is important in the progression of late-stage CRCs (Kataoka *et al*, 2004). Interestingly, decreased EphA7 and EphA6 expression has been reported in pooled CRCs compared to normal colon (Hafner *et al*, 2004). This finding was confirmed in CRCs where epigenetic silencing was shown to be associated with the downregulation of EphA7 (Wang *et al*, 2005).

**Figure 7:**
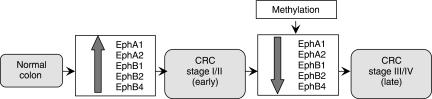
Pathway in colorectal cancer (CRC) progression from normal colon to late-stage cancer.

An interesting observation emerging from these studies is the likely potential for these genes to have separate yet overlapping function in CRCs. Our data and other studies suggest that one or more of the Eph genes, in particular, *EphA1*, *EphA2*, *EphB1*, *EphB2* and *EphB4*, are upregulated and may have an oncogenic function in the early stages of malignant transformation in the colon (Figure 7). Subsequent gene silencing through methylation and/or somatic genetic changes may be important in facilitating tumour migration/invasion. Given the direct correlation between loss of EphA1 expression and the progression of CRC to a more invasive phenotype, EphA1 appears to be a potentially valuable marker, particularly as part of algorithms for defining those patients with a poor prognosis.

Eph proteins have been identified as therapeutic targets in cancer, both as anti-cancer and anti-angiogenic agents. Several therapeutic candidates including antibodies to EphA2 (Landen *et al*, 2005), EphA3 (Vearing *et al*, 2005) and EphB2 (Mao *et al*, 2004) are in advanced pre-clinical or early clinical assessment. Although therapies targeting the high EphA1 expression in early phase CRC seem logical, the loss of expression in advanced disease poses the risk that targeted therapies may select for loss of expression and thus contribute to disease progression. Recognising the surprising biphasic pattern of EphA1 expression during CRC progression, and potentially in other epithelial tumours, requires a careful evaluation of the function of Eph expression in CRC and their rational targeting with Eph-specific therapies.
