# Supplementary material for: Epigenetic silencing of EphA1 expression in colorectal cancer is correlated with poor survival
Source: Br J Cancer. 2009 Mar 10;100(7):1095–102. doi: 10.1038/sj.bjc.6604970 (PMC2670002; doi:10.1038/sj.bjc.6604970)
Supplement: Supplementary methods [file 6604970x1.doc]

**SUPPLEMENTARY ONLINE MATERIAL**

#### Supplementary Methods

#### Immunoprecipitation and Western analysis

Cells were lysed (150mM NaCl, 25mM Tris-Cl pH 6.8, 1% Triton X-100, 1mM Na_3_VO_4_, 4mM NaF and protease inhibitors) and immunoprecipitated with 10μg of an in-house affinity purified polyclonal rabbit EphA1 antibody (minimum 2 hours at 4°C), then precipitated with protein A-Sepharose beads (Sigma). The beads were resuspended in SDS sample buffer (125mM Tris-Cl pH 6.8, 4% w/w SDS, 100mM DTT, 20% glycerol and 0.01% bromophenol blue) and subjected to 7.5% SDS polyacrylamide gel electrophoresis (SDS-PAGE). The gels were transferred onto nitrocellulose membrane (Amersham) and blocked with 5% non-fat dry milk in TBST (50mM Tris-Cl pH 8, 150mM NaCl, 0.02% Tween 20). EphA1 protein was detected with a polyclonal antibody against EphA1 (R&D systems, Australia). After washing with TBST, secondary peroxidase conjugated antibody was added (BIORAD, Hercules, CA). Immunoreactive bands were visualised using ECL™ substrate (Amersham, Piscataway, NJ).

**Immunohistochemistry**

Paraffin sections (3-4μm) were dewaxed in xylene and rehydrated to water through descending graded alcohols. Heat antigen retrieval was performed by incubating in 0.01M citrate buffer (pH 6) at 90°C for 40 minutes. After cooling, the sections were transferred to Tris buffered saline (TBS; 0.1M Tris-Cl pH 7.4, 0.15M NaCl), pH 7.4. Endogenous peroxidase activity was quenched by incubating the sections in 1% H_2_O_2_, 0.1% NaN_3_ in TBS for 10 minutes. All blocking and binding incubations occurred in a humidified chamber. After a brief wash in TBS, the sections were blocked with 10% non-immune donkey serum for 20 minutes.

Excess serum was decanted and the sections were incubated overnight with a goat polyclonal EphA1 antibody (R&D systems, Australia) (10µg/ml in TBS). The sections were washed in TBS and incubated with horseradish peroxidase conjugated donkey anti-goat immunoglobulins (Biocare Medical, Australia) for 45 minutes. Antigenic sites were revealed using 3,3'-diaminobenzidine tetrahydrochloride (Sigma Aldrich, St Louis, MO) with H_2_O_2_ as substrate in TBS, counter-stained with haematoxylin and mounted using DePeX (BDH Gurr, Poole, UK).

The intensity of EphA1 staining in each tumour sample was assessed by comparison with the adjacent non-malignant colon.

**Flow Cytometry**

All washes and antibody binding steps used 5% FBS in PBS to reduce non-specific protein binding. 1x10^5^ cells were washed three times and incubated with a primary rabbit anti-EphA1 antibody (in house) for 30 minutes at room temperature. The cells were washed twice and incubated for 15 minutes with an anti-rabbit FITC (Chemicon, Australia) at room temperature in the dark. After several washes the cell pellet was re-suspended in propidium iodide in PBS (20µg/ml). Cell fluorescence (two-color) was detected using a BD FACSCalibur™ and data acquired using CellQuest Pro v4.0.2. Subsequent analysis of fluorescence data was conducted using Summit® v3.1.

**Relative Quantitation by Real-Time PCR**

Quantitative real-time PCR (QPCR) was carried out using Quantitect™ SYBR® Green PCR Master Mix (QIAGEN, Australia) following the manufacturer’s instructions. For tissue arrays, 1 x Quantitect™ SYBR® Green PCR Master Mix was added to 13 μl of ddH_2_O. Forward and reverse primers were added to a final concentration of 0.66 μM per reaction. Q-PCR was carried out in an ABI Prism 7900HT thermocycler (Applied Biosystems, USA).

For cell lines and CRC clinical samples, 5 μl of diluted cDNA was added to Quantitect™ SYBR® Green PCR Master Mix. Forward and reverse primers were added to a final concentration of 0.3 μM. β-actin was chosen in this instance as the house keeping gene as this showed a good coefficient of correlation for colorectal tissues in accord with other reports (de Kok *et al*, 2005). QPCR primer sequences are listed in supplementary Table 2. All reactions were performed in duplicate to assess reproducibility. QPCR was carried out in a Corbett Research Rotor-Gene 3000™ (Corbett Research, Australia). The PCR cycling conditions included activation for 15 minutes at 95ºC and 40 cycles of 30 seconds at 95ºC, 30 seconds at 60ºC, and 30 seconds at 72ºC. Fluorescence data was recorded at the end of each 72ºC step. A DNA melt profile was run subsequently from 72ºC to 95ºC with a ramp of 1ºC/5 seconds. Fluorescence data was recorded continuously during the melt profile.

Copy number analysis relative to β-actin was used to assess tissue arrays and the Pfaffl method of quantification was used for data analysis of the paired normal and tumour samples (Pfaffl *et al*, 2001). Similar to the standard curve method, the Pfaffl method provides a means for quantification of a target gene transcript in comparison to a reference gene. The relative expression ratio for EphA1 was calculated using QPCR efficiencies and the crossing point deviation of an unknown sample versus an internal control (calibrator). Unlike the standard curve method this model needs no calibration curve, as control levels are included within the model.

**SUPPLEMENTARY RESULTS**

**Supplementary Figure 1** EphA1 immunohistochemistry

C

D

Panels A and B represents two stage II CRCs with strong EphA1 expression Membranous and cytoplasmic staining of EphA1 is detected in both cases. Panels C and D represent paired normal colonic epithelium and a stage III colon cancer. (C) Lateral membrane staining is prominent at the surface epithelium and is absent towards the base of the crypt. (D) No EphA1 expression was detected in the corresponding tumour.

**Supplementary Figure 2** Region of EphA1 genomic sequence containing CpG island

Human EphA1

ccccgtctctactaaaaatagaaaaattagctgggtgtgg
tggtgcacgcttgacgcttgtgatcccagttactcgggaggctgaggcaggaggatcgtt
tgaaccagggaggcggaggttgcagtgagccaagattgcaccactgcactgcagcctggg
ccacagagcgagactccatctcaaaaaagaaaaagaatataaggaagtccgcaagaaagt
gagagggcacgtggtttgacaccactaagatgggttaaaatatccttccctgccacctgt
ggctggaaccttaaccagatattcatgtctgctgtgtaagtgcatgtcaaggttcaaact
ccttttcttgtttacccagccctggcccacacctgcctttctgccctgattcccaagaca
aatcctgcgccccagcagtcaaactggtccattcaccactccctgggcgcaccttgaggc
tccctgccttcaggcccttgctggtgcctttcccaatctctgccactgaaaacccaggtc
acaccagctctccctgaagactcaactaatgtgccccgatgactgggcttgcttcctctt
ctaatctccagatgcttcactgcaggttgtaccccaccttgcattgtggtcatctaagcc
tcccctgctagagggtgaagatatgactcttaccccttgccttccccggccctattaccc
agctctggggcctgcacaaagacagaaagaggaatataggagtgtaattcaagtcagagg
tagaattaggttaaaagctggatcaagattgggttgagtttaggattagaattgggaaaa
ggttcgtcaattcttaatcccacctactaggcggcgcccgcagtctcattcgggtcaaga
cgcctccggttcttgaatttgattaccagtctagcaagtttctttcctgccctactgtcc
ctttaaggaggtgaaccaggtgaggccgggactccgcccccggcgctggccccgcccccc

GCCCCCGCCCGGCCCGCCCGGCTCTCCTAGTCCCTTGCAACCTGGCGCTGCCATCCGGGC
CACTGTCCCAGGTCCCGGCCCGGAGCT***ATG***GAGCGGCGC*TGGCCCCTGGGGCTAGGGCTG*
*GTGCTGCTGCTCTG****CG****CCC****CG****CTGCCCC****CG****GGGG****CGCGCG****CCAAGGAAG*

*gtac****cg****acccc****cg****cccc****cgcg****cccccaacccc****cgcg****ccc****cg****ccccttcc****cg****gggcc****cg****gc
cagcacccccctgcagaagc****cg****gcattcctaggggccatgttccc****cg****agaccct****cg****ccag
ctctct****cg****ctgtgcc****cg****ac****cg****ccaggctat****cg****actggccaggcccatctttggtctttaa
c*cccaagcggctgctctcccgggtcagtcacccccactcccgcaggccagcaatctgctc
ctgggagtggggagggaattccggacccttgccaggaagtagcacctgcacccgcacccc
tgcctgcattcccagacagtcccagcttcaggtgcccaccccagagatctgagcccttgg
aagttgaaggtgggaagtcgcctcctgccaacctaggccagggacagagggagctgcctc
cgggagaagggttaggactgacgccacaggggttcccagttgtccgagtcgaatattcca
tgtgaaagttggaacaccctctccggccttactcccccccacttcccaggcaggtagctg
tgcctcttatccccacttcacggctctgatcactggccaggtcaggccaaagcaggcttc
ggacagtggaggtgagcctccctacccaggaaaccccttctgaggtccccggtccaccct
gcccactcctggctagcagaggtgagctcctcctgccctccacgcgttccaatcctgcag
ctgatgggtgtcccaggacgcagcagcctgggtaactgagcgtctcgtggggctgctctt
cttgcattgactttggtcaaagacccagcgccccaacccttgtcccactctacctgcctt
ccttcacacacacaaagaagagtgaaatccctggagctcttcactcctcagtcctgagcc
ctttttctcccctaagcctcaggaagtcacagcttttaggaacaggatgttgcagcctgg
ggctgaggccccagaatgattaatgtgggctgcaggggcacctcctcccccagaggtgac
agacttcttttctccccactttcag

TTACTCTGATGGACACAAGCAAGGCACAGGGAGAGCTGGGCTGGCTGCTGGATCCCCCAA
AAGATGGG

The first 1000bp represent the 5′ flanking sequence from the transcription start site, or if this has not been identified, from Met_i_. The 5′ UTR region is represented in gray upper case letters which are underlined. This is followed by exon 1 (coding portion) in upper case letters and intron 1 (non-coding region) in lower case letters followed by exon 2 in upper case letters. CpG island starts 13bp downstream of the translation start site where the A of the ATG was considered +1 and spans exon 1 and intron 1. This region is italicised and underlined. The CG/cg CpG dinucleotides within the island are further highlighted in gray, bold letters.

**Supplementary Figure 3** CPGPLOT island of unusual CG composition in human EphA1

This figure represents that the observed/expected CG ratio was greater than 0.60 and the percent C + G was greater than 50 in the region highlighted between nucleotide 1100 to 1350. This numbering refers to the genomic sequence of EphA1. These criteria fit the acceptable definition of a CpG island.

**Supplementary Figure 4** DNA melt profile of EphA1


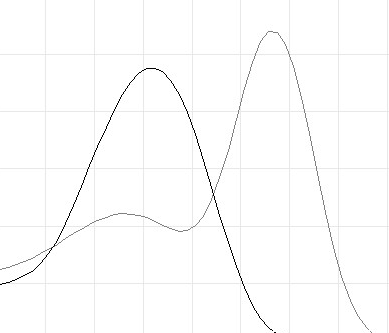


A

LIM1215

Tm=83.25

*Sss*I treated

LIM1215

Tm=85.75


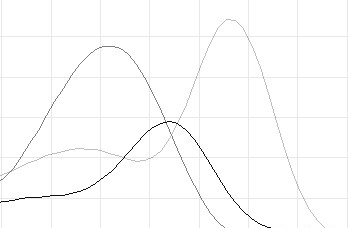


B

*Sss*I treated

LIM1215

Tm=85.75

Clinical Sample

LIM1215

Tm=83.25

DNA melting profile of the EphA1 CpG island for LIM1215 as determined by melt curve analysis. LIM1215 had no detectable methylation in its unmodified state, however upon *Sss1* methyltransferase treatment complete methylation of the genomic DNA was detected. This resulted in a shift of the Tm from 83.25ºC to 85.65ºC.

**Supplementary Figure 5** EphA1 methylation

A

B

(A): Dot plot representing the distribution of EphA1 expression in methylated and unmethylated groups (determined by bisulfite sequencing and melt curve analysis). Long horizontal lines represent the mean and short lines the standard deviation. (B): Percent methylation observed in the entire CpG island and the 3′ end of the CpG island determined through bisulfite sequencing.

**Supplementary Table 1**

| **Tumours** | **Sex** | **Age** | **^*^TNM Stage** | **^†^ Grade** | ‡**Type** | **^§^Site** |
| --- | --- | --- | --- | --- | --- | --- |
| **1** | F | 74 | III | 3 | 2 | 2 |
| **2** | F | 52 | III | 3 | 1 | 2 |
| **3** | M | 73 | II | 2 | 1 | 5 |
| **4** | F | 56 | III | 2 | 1 | 2 |
| **5** | M | 69 | III | 2 | 2 | 2 |
| **6** | F | 77 | II | 2 | 1 | 2 |
| **7** | M | 77 | III | 2 | 1 | 4 |
| **8** | M | 76 | II | 2 | 1 | 5 |
| **9** | F | 72 | II | 2-3 | 1 | 6 |
| **10** | M | 76 | III | 2 | 1 | 5 |
| **11** | F | 79 | II | 2 | 2 | 1 |
| **12** | M | 72 | IV | 2 | 1 | 6 |
| **13** | M | 79 | III | 2 | 1 | 2 |
| **14** | M | 68 | II | 2 | 1 | 5 |
| **15** | M | 64 | II | 2 | 1 | 1 |
| **16** | F | 78 | II | 3 | 1 | 3 |
| **17** | F | 65 | II | 2 | 1 | 2 |
| **18** | M | 46 | II | 2 | 1 | 6 |
| **19** | M | 79 | III | 2 | 1 | 5 |
| **20** | F | 29 | II | 2 | 1 | 3 |
| **21** | M | 43 | III | 3 | 1 | 5 |
| **22** | F | 77 | II | 2 | 1 | 3 |
| **23** | M | 79 | III | 3 | 1 | 5 |
| **24** | F | 57 | III | 2 | 1 | 2 |
| **25** | M | 90 | III | 3 | 2 | 2 |
| **26** | M | 77 | III | 2 | 1 | 5+6 |
| **27** | M | 66 | III | 2-3 | 1 | 4+5 |
| **28** | M | 83 | IV | 2 | 1 | 1 |
| **29** | M | 58 | III | 3 | 1 | 2 |
| **30** | F | 80 | II | 2 | 2 | 2 |
| **31** | F | 61 | II | 2 | 1 | 2 |
| **32** | F | 60 | III | 2-3 | 2 | 3 |
| **33** | F | 81 | III | 3 | 1 | 1 |
| **34** | F | 76 | II | 3 | 2 | 2 |
| **35** | F | 74 | II | 2 | 2 | 1 |
| **36** | F | 72 | II | 3 | 2 | 4 |
| **37** | F | 67 | IV | 3 | 2 | 2 |
| **38** | F | 67 | N/A | N/A | N/A | N/A |
| **39** | F | 75 | II | 2 | 1 | 6 |
| **40** | M | 75 | I | 2 | 2 | 6 |
| **41** | M | 50 | II | 2 | 1 | 1 |
| **42** | M | 70 | II | 2 | 1 | 1 |
| **43** | M | 67 | I | 2 | 1 | 5 |
| **44** | M | 35 | I | 2 | 1 | 6 |
| **45** | M | 75 | II | 2 | 1 | 6 |
| **46** | M | 69 | III | 2 | 1 | 9 |
| **47** | F | 66 | II | 2 | 1 | 2 |
| **48** | F | 62 | II | 2 | 1 | 2 |
| **49** | F | 77 | II | 2 | 2 | 6 |
| **50** | M | 78 | II | 2 | 1 | 2 |
| **51** | M | 57 | III | 2 | 1 | 2 |
| **52** | M | 57 | I | 2 | 1 | 6 |
| **53** | F | 84 | II | 2 | 1 | 9 |

No information other than age and sex no is available for patient 38

**^*^** TNM staging system was used to characterise these tumours (see text for details)

**^†^** 2 refers to moderately differentiated whereas 3 refers to poorly differentiated

‡ Type: 1 refers to adenocarcinomas whereas 2 refers to mucinous adenocarcinomas

**^§^** Site: 1-caecum, 2- ascending colon, 3-transverse colon, 4-descending colon, 5-sigmoid, 6-rectum, 9 not available

**Supplementary Table 2**

Primers used in QPCR

| Gene  (Acc. No.) | Forward Primer (5′ – 3′) | Reverse Primer (5′ – 3′) |
| --- | --- | --- |
| EphA1 (NM_005232) | GTGGACACTGTCATAGGAGAAGG | GGTCTTAATGGCCACAGTCTTG |
| β-actin (NM_001101) | GACTCAACACGGGAAACCTC | AGCATGCCAGAGTCTCGTTC |

Genbank Accession Numbers are listed in parenthesis.

Supplementary Table 3

| **Tumours** | **EphA1 copy number**  **(per 1000 copies of beta-actin)** | **Immunohistochemistry** |
| --- | --- | --- |
| **1** | 2.95306 | Positive |
| **2** | 3.258359 | Positive |
| **3** | 3.717784 | Positive |
| **4** | 4.686997 | Positive |
| **5** | 2.353239 | Positive |
| **6** | 4.214918 | Positive |
| **7** | 3.124213 | Positive |
| **8** | 4.387314 | Positive |
| **9** | 1.659145 | Positive |
| **10** | 1.093213 | Positive |
| **11** | 0.011032 | Negative |
| **12** | 0.063903 | Negative |
| **13** | 0.189587 | Negative |
| **14** | 0.042423 | Negative |
| **15** | 0.852857 | Negative |
| **16** | 0.864856 | Weakly positive |
| **17** | 0.163969 | Negative |
| **18** | 3.960586 | Negative |
| **19** | 0.347331 | Positive |
| **20** | 0.575312 | Negative |
| **21** | 15.95625 | Positive |
| **22** | 6.730705 | Positive |
| **23** | 0.225729 | Weakly positive |
| **24** | 4.697309 | Negative |
| **25** | 4.996266 | Positive |
| **26** | 6.187505 | Positive |
| **27** | 1.04439 | Weakly positive |
| **28** | 0.92659 | Weakly positive |
| **29** | 1.986075 | Weakly positive |
| **30** | 0.066334 | Negative |
| **31** | 0.511146 | Negative |
| **32** | 11.57316 | Positive |
| **33** | 0.351778 | Negative |
| **34** | 4.387009 | Positive |
| **35** | 1.952653 | Weakly positive |
| **36** | 2.413711 | Positive |
| **37** | 0.518335 | Negative |
| **38** | 1.47368 | Positive |
| **39** | 3.316269 | Positive |
| **40** | 5.435478 | Positive |
| **41** | 0.926399 | Comparable |
| **42** | 0.476323 | Negative |
| **43** | 0.136653 | Negative |
| **44** | 2.526297 | Negative |
| **45** | 0.058968 | Negative |
| **46** | 1.831362 | Weakly positive |
| **47** | 0.494997 | Negative |
| **48** | 1.333508 | Positive |
| **49** | 2.687403 | Positive |
| **50** | 2.371578 | Positive |
| **51** | 0.773907 | Comparable |
| **52** | 0.204086 | Negative |
| **53** | 1.053366 | Positive |
